# Early surgical and oncological outcomes during adoption of a single port VATS lung resection in a tertiary cancer center: a retrospective analysis

**DOI:** 10.1186/s13019-022-01777-y

**Published:** 2022-03-02

**Authors:** Riad Abdel Jalil, Mohamad K. Abou Chaar, Obada Al-Qudah, Ahed Al-Edwan, Omar Almajali, Hazim Ababneh, Ahmad U’wais, Munir Al-Ghazawi, Hani Al-Najjar, Ahmad Abu-shanab

**Affiliations:** 1 Department of Thoracic Oncology, King Hussein Cancer Center, Queen Rania Al Abdullah Street, P.O. Box 1269, Amman, 11941 Jordan; 2 Department of Surgery, King Hussein Cancer Center, Amman, Jordan; 3 Department of Anesthesia, King Hussein Cancer Center, Amman, Jordan; 4 Department of Research, King Hussein Cancer Center, Amman, Jordan

**Keywords:** Single port VATS, Lobectomy, Complex lung resection, Lung cancer, Pulmonary metastasectomy

## Abstract

**Background:**

Video-assisted thoracoscopic surgery (VATS) is a safe and effective surgical approach for pulmonary resection. VATS can be accomplished with only a single incision, resulting in less postoperative pain and paresthesia, better cosmetic results, and greater patient satisfaction. Single-port VATS (spVATS) has become increasingly common for lung resection. We assess the early surgical and oncological outcomes after adopting this new technique at our tertiary cancer center as the first institution to do so in the country.

**Method:**

Medical records for 257 patients in a tertiary cancer center, with a diagnosis of non-small cell lung cancer, pulmonary metastasis, or other chest-confined pathology, were accessed to obtain perioperative outcomes, pathologic results, post-operative follow-up data, and early surgical and oncological outcomes. All patients underwent spVATS for limited or major lung resection. Simple descriptive analysis was utilized.

**Results:**

spVATS was either performed with curative intent (79.8%, N = 205), or as a diagnostic procedure (20.2%, N = 52). Resection types were subcategorized for curative intent group as limited (73.6%, N = 151), lobectomy (16.6%, N = 34), and complex (9.7%, N = 20). Resection with a negative margin (R0) rate was 100% for the primary lung cancer (PLC) patients and 97% for the pulmonary metastasectomy (PM) group. The complication rate was 5%. Three-year disease-free survival was 87% and 68.5% for PLC and PM group, respectively. The 3-year overall-survival was 91.3% for the PLC and 82.8% for PM. Operation duration showed a downtrend over the study period in each curative subcategory with a borderline difference in the limited resection (*P* value = 0.05).

**Conclusion:**

All the spVATS procedures were successfully performed without perioperative severe complications or mortality, regardless of complexity. R0 resection was excellent. Middle- and long-term efficacies of spVATS for lung cancer require further follow-up. With proper training, appropriate indication and meticulous application, adopting spVATS is safe and feasible technique that does not compromise surgical and oncological outcomes.

**Supplementary Information:**

The online version contains supplementary material available at 10.1186/s13019-022-01777-y.

## Background

The video-assisted thoracoscopic surgery (VATS) technique is the individual dissection of veins, arteries, and lobar lung bronchi, together with mediastinal lymphadenectomy, using a video thoracoscopic approach visualized on-screen and involving 1 to 4 incisions or ports in the absence of rib spreading [1]. It has been suggested that thoracoscopic lung surgery is associated with reduced postoperative pain compared with conventional thoracotomy [2]. According to the review published by Coffey et al., lung resections performed on a minimally invasive basis could favorably influence oncological outcomes obtained [3].

The surgical evolution in the VATS approach from three-ports to the two-port technique required a different lung exposure and learning how to move the instruments during surgery. However, the final step of the surgical evolution in the VATS approach to minimize chest wall trauma was the single-port approach, described by Rocco et al. for wedge pulmonary resection [4]. The latter approach was further developed by Gonzalez-Rivas et al. to enable major pulmonary resections [5].

The technical feasibility, steep learning curve, and excellent early outcomes of the 'ultra-minimally invasive’ single-port VATS (spVATS) have allowed many experienced thoracic surgeons to adopt this approach quickly. Furthermore, with the expert thoracic surgeons' meticulous skills and innovative minds, spVATS has evolved and branched into many fascinating niches [6].

It has been shown that the spVATS approach has less postoperative pain, faster recovery, better cosmesis, and preserving a good hilar exposure and the ergonomics of the procedure with a small incision [7]. Liu et al. confirmed that the total number of lymph nodes dissected via spVATS could be even higher than conventional VATS, suggesting that the single-port technique does not compromise lymph node dissection [8]. Our study was designed to assess the safety and early oncological outcomes after adopting this approach at King Hussein Cancer Center (KHCC).

## Methods

A single institution retrospective study design with inclusion criteria of all adult patients who underwent spVATS between January 2017 and August 2020, who were operated on by the same senior thoracic surgeon. The study proposal was reviewed and approved by the Institutional Review Board at KHCC (19 KHCC 139). Medical records were accessed to collect pathological diagnosis, type of surgery, operative time, postoperative complications, length of stay, and last follow-up date associated with the state of disease for each patient. The patients were divided into those who underwent spVATS for curative intent and those for diagnostic purpose. The latter category entailed performing a lung biopsy.

Surgical resection for curative intent was further categorized into (1) complex, (2) lobectomy, and (3) limited resection. Complex procedure was identified as any patient who underwent (a) bilobectomy, (b) lobectomy with chest wall resection, (c) lobectomy with pericardial resection, (c) lobectomy with atrial resection, or (d) any form of resection after neoadjuvant therapy. Limited subgroup included patients who underwent segmentectomy or wedge resection. Operative time was calculated from the moment of the initial incision until closure of the surgical wound.

Descriptive data analysis was performed using IBM^®^ SPSS^®^ Modeler 16.0 (International Business Machines Corporation, USA). Median operative time in minutes for the three subcategories was plotted against each year of the study period using Independent-Samples Kruskal–Wallis Test.

### Operative technique

Under general anesthesia, single-lung ventilation was performed using a double-lumen endotracheal tube or endobronchial blocker. The patient was placed in a full lateral decubitus position with flexion of the operating table at the level of the scapula tip to widen the intercostal space and get better chest exposure. A paravertebral and shoulder block is usually done just before the skin incision. A single incision of approximately 4 cm was created in the 5th intercostal space a few centimeters medial to the anterior edge of the latissimus dorsi muscle, providing good access and control to the lung hilum. The operating surgeon and his assistant were usually positioned to face the patient's front to simulate the field and direction of vision. A small-sized wound protector (2.5–6 cm) was utilized to minimize contamination and expand the incision. We used the 30-degree lens scope placed posteriorly in relation to the access incision with the anterior portion's instruments. For tissue handling, long curved thoracoscopic devices were utilized for manipulation, suction, and dissection. Long shaft endo-staplers with small reloads (35–45 mm) for vascular resection, and 60 mm reloads for bronchial and parenchymal resection. The specimen is retrieved by endo-bag to minimize wound contamination and seeding. All patients Underwent shoulder block as well as paravertebral nerve block in three levels; at the wound level, one intercostal space above and one below using Bupivacaine 0.5% with the dose given calculated according to the patient's weight not exceeding the maximum dose. We do the nerve block after the positioning and before the incision.

The chest tube was placed at the anterior edge of the wound and connected to the underwater seal device with negative suction. Skin was sutured in multiple layers (Additional file 1A, B).

## Results

**Fig. 1:**
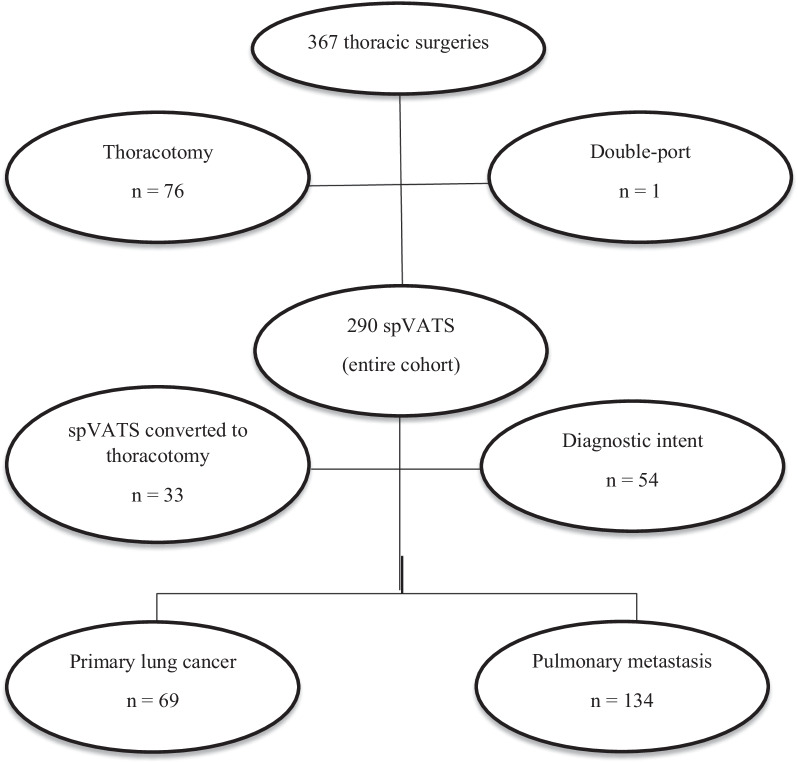
Consort diagram detailing inclusion and exclusion criteria with description of each group studied. Consort diagram showing all patients who underwent spVATS for curative intent, between January, 2017 and August, 2020. Diagnostic procedures and operations that required conversion were excluded

A total of 291 patients underwent spVATS. Due to intra-operative findings, 33 (11.3%) cases were converted to open thoracotomies and 1 (0.3%) case was converted to double-port VATS with a conversion rate of 11.7%. A total of 54 (18.5%) patients underwent diagnostic procedures. spVATS was done for curative intent in 203 (69.7%) patients, of which 69 (34%) had a diagnosis of primary non-small cell lung cancer (PLC) and 134 (66%) had pulmonary metastasis (PM) (Fig. 1). PLC and PM were further divided according to the type of resection into: limited resection (28, 40.6% and 120, 89.5%), lobectomy (28, 40.6% and 7, 5.2%), and complex procedure (13, 18.8% and 7, 5.2%). The latter group included patients who underwent bronchial sleeve upper lobectomy, bilobectomy, lung resection enblock with chest wall, diaphragm or pericardium, and one case with resection of a small part of the left atrium.

The majority of patients in both groups did not have postoperative complications, with only 3 (4.3%) patients in the PLC group having pulmonary complications, 1 (1.4%) patient suffered from atrial fibrillation, and 2 (2.9%) had infection requiring intravenous antibiotics. On the other hand, 2 (1.5%) patients in the PM group had cardiac complications and 2 (1.5%) had postoperative infection. The rate of negative resection margin (R0) was 97% and 100% for the PM group and the PLC group, respectively. The median length of stay was 3 days for PLC patients and 1 day for PM resection. Three-year disease-free survival was 68.6% and 87%, and the overall survival was 82.8% and 91.3% for PM and PLC groups respectively (Table 1).

**Table 1:** Simple descriptive analysis showing the complication rate, margin status, length of stay, and survival rate between the primary lung cancer group and the pulmonary metastasis

	Primary lung cancer (69, 34%)	Pulmonary metastasis (134, 66%)
Surgical complexity		
Limited	28 (40.6%)	120 (89.5%)
Lobectomy	28 (40.6%)	7 (5.2%)
Complex	13 (18.8%)	7 (5.2%)
Complications		
None	63 (91.3%)	130 (97%)
Pulmonary^a^	3 (4.3%)	0
Cardiac^b^	1 (1.4%)	2 (1.5%)
Infection^c^	2 (2.9%)	2 (1.5%)
Margin		
Positive	0	4 (3%)
Negative	69 (100%)	130 (97%)
LOS^d^ (days)		
Mean (± SD^e^)	3.67 (± 2.801)	1.9 (± 2.084)
Median (Min–Max)	3 (1–14)	1 (1–20)
Survival rate		
DFS^f^	87.0%	68.6%
OS^g^	91.3%	82.8%

^a^Laryngeal edema, emphysema, and chyle leak

^b^Cardiac arrest and atrial fibrillation

^c^Surgical site infection, empyema, lung consolidation

^d^Length of stay

^e^Standard deviation

^f^Disease free survival

^g^Overall survival

**Fig. 2:**
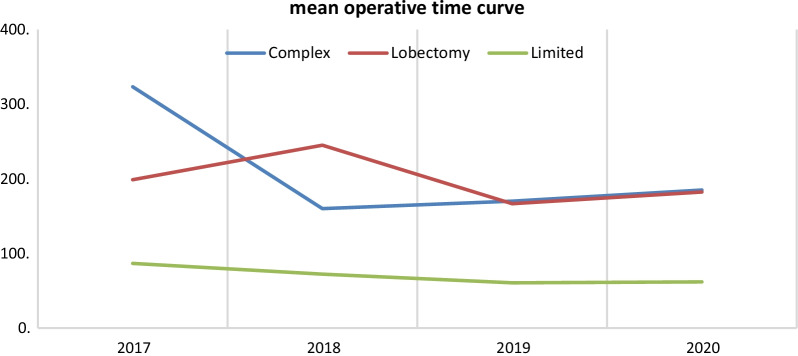
Mean operative time in minutes (x-axis) plotted against time in year (y-axis) between the period 2017 and 2020 for each curative spVATS subcategory. MOT curve

**Fig. 3:**
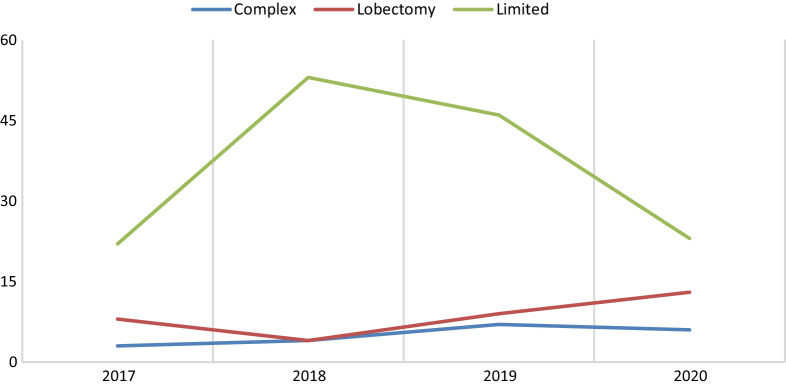
Number of spVATS (x-axis) plotted against time in year (y-axis) between the period 2017 and 2020. Number of spVATS against time

Initial observation of the mean operative time (MOT) curve showed improvement between 2017 and 2020 in all subcategories. Complex resection MOT was of 323.2 min decreasing to185 minutes, lobectomy group had a MOT of 198.75 min and became 182.3 min, and limited resection group had a MOT of 86.6 min in 2017 and 61.9 min in 2020 (Fig. 2). Due to an unequal and small sample size in some subcategories (Fig. 3), further analysis was made using Kruskal–Wallis test that showed insignificant difference between the MOT, in each of the complex and lobectomy groups, and duration of the study. *P* value was borderline in the limited resection group (*P* value = 0.05) that showed on a pairwise comparison a significant difference between the years 2017 and 2019 (*P* value = 0.039) (Tables 2, 3).

**Table 2:** Independent-samples Kruskal–Wallis test comparing the mean operative time for the curative intent subgroups over the study timeline

Complex	Total N	20
	Test statistic	5.182
	Degree of freedom	3
	Asymptotic sig. (2-sided test)	0.159
Lobectomy	Total N	34
	Test statistic	2.253
	Degree of freedom	3
	Asymptotic sig. (2-sided test)	0.522
Limited	Total N	151
	Test statistic	7.818
	Degree of freedom	3
	Asymptotic sig. (2-sided test)	0.050

**Table 3:** Pairwise comparisons of year in the limited resection group

Type	Sample 1–Sample 2	Test statistic	Std. error	Std. test statistic	Sig.	Adj. Sig.*
Limited	2019–2020	− 5.217	11.103	− 0.470	0.638	1.000
	2019–2018	12.127	8.531	1.422	0.155	0.931
	2019–2017	30.659	11.271	2.720	0.007	0.039
	2020–2018	6.910	10.726	0.644	0.519	1.000
	2020–2017	25.442	13.012	1.955	0.051	0.303
	2018–2017	18.532	10.900	1.700	0.089	0.535

Each row tests the null hypothesis that the Sample 1 and Sample 2 distributions are the same in regards to the mean operative time

*Significance values have been adjusted by the Bonferroni correction for multiple tests

## Discussion

Ever since it was first described in 2004, spVATS for lung neoplasm management has been developing rapidly [9]. In this study, we analyzed the characteristics of 257 patients, from a clinicopathological and surgical aspect, all of whom underwent spVATS for lung biopsy, lung wedge resection, segmentectomy, lobectomy, bilobectomy, lung resection for locally advanced tumors, lung resection after neoadjuvant chemotherapy, and lung resection enblock with the chest wall, diaphragm, pericardium or a small part of the left atrium. The high rate of negative resection margin, a short period of hospital stay, and a low rate of complications proved the efficacy of spVATS in cancer patients.

One of the earliest published case series during the last decade included 97 patients who underwent spVATS for lobectomy, segmentectomy, or pneumonectomy reported the safety of major lung resection with a 2.9% conversion rate to open thoracotomy and without any mortality [5]. A cohort published by Ng et al. that included 150 patients; 87% had nonsmall-cell lung cancer, 6% had other lung malignancies, and the rest had either pulmonary metastasis or benign lesions, reported a higher rate of conversion to double port VATS (5.3%) which they attributed to vascular, or anatomical complications. They indicated that due to the high prevalence of tuberculosis in Asia, extensive pleural adhesions could be encountered intraoperatively. They further added that small stature and rib cage size, resulting in a narrow intercostal space, restricted intrathoracic area for manipulation, and increased incidence of instrumental fencing, are encountered frequently in the Asian population, and they confirmed the safety of spVATS in the targeted population with a disease-free survival rate of more than 80% regardless of stage [10].

Wu et al. indicated that spVATS had better outcomes in cosmesis, recovery time, and pain when utilized for non-invasive mediastinal tumor resection [11]. This was followed by two comparative studies, the first done by Song et al., which demonstrated better outcome in mediastinal lymph node dissection but failed to detect any difference between spVATS and triple-port VATS regarding operative time, intraoperative bleeding, and rate of conversion. Wang et al. showed a significant difference in intraoperative time and operative blood loss between spVATS and double/triple-port VATS in favor of the former group [12, 13]. Based on preliminary and unpublished KHCC data, the incidence of shoulder pain after thoracic surgery, mainly attributed to the lateral position and shoulder extension for better exposure during surgery, went down from 50% in patients to less than 10% after utilizing the combined paravertebral and shoulder block.

Multiple studies emphasized the importance of practice in acquiring the needed skills for spVATS, knowing that a transition from multiple-port VATS is easier than open thoracotomy [14–16]. The primary investigator, who is the senior thoracic surgeon, started performing triple-port VATS transitioning to double-port VATS, followed by extensive training in October 2016 at Shanghai Pulmonary Hospital with a group of pioneers in spVATS. After which we started to utilize this technique at KHCC. It was expected to notice an increase in number of procedures performed over the study timeline period, which was evident in all curative extent subcategories (Fig. 2). Due to the unpredictability and anatomical variation encountered during complex spVATS and lobectomy procedures, MOT did not show a significant decrease over time. In the limited resection subgroup, the most significant difference was most evident between the years of 2017 and 2019 followed by 2017 and 2020 but after Bonferroni correction, the latter difference was redeemed insignificant, a finding that can be attributed to the fact that they study period did not include all of 2020. Due to COVID-19 pandemic, and the emerging data supporting higher postoperative mortality rate after intubation [17], KHCC adhered to the international recommendations and reduced the numbers of cases. Such implementation, resulted in a sharp decrease in cases of limited resection and slight reduction in complex cases as compared to the previous years—(Fig. 3).

One of the technical issue discussed at length was the limitation of the visual field during spVATS; this was solved by utilizing a rotating prism mechanism at the tip of a rigid scope allowing vision between 0° and 120°. A more advanced flexible scope has been suggested by Yang et al. utilized in right upper lobectomy, which was criticized due to the soft scope's delicate tip [18]. Both techniques might be replaced by the recently developed magnetic anchoring and guidance system (MAGS) camera that could result in a larger viewing angle and eliminate instrument fencing [19].

Proper selection of patients, having the instruments suitable for this approach and knowing when to switch to multiple port or even open thoracotomy to ensure patient's safety as well as adherence to the best oncological rules enable us to adopt this approach without compromising the surgical nor the oncological outcomes for our cancer patients.

This study had the following limitations; retrospective and single centered, no comparison was made between multiport and spVATS, neglecting demographic variables, and inclusion of cancer patients only. It must be highlighted that the same senior surgeon did all surgeries, which enabled comparing the mean operative time for the curative spVATS groups.

## Conclusion

We have reviewed the feasibility, safety, and oncological outcomes for lung resection as a diagnostic and a curative approach for both primary lung cancer and pulmonary metastasis after adopting a spVATS technique, performing a fair amount of major and complex procedures. There was no perioperative severe complication or mortality, regardless of complexity of resection. The immediate oncological outcome in terms of complete R0 resection was excellent. The middle- and long-term effectiveness of this technique in treatment of lung malignancy still requires further follow-up. spVATS is just another variant of video-assisted thoracoscopic surgery in the modern era that can benefit patients compared with conventional techniques, resulting in less trauma and less postoperative pain without compromising oncological outcomes.

## Supplementary Information

**Additional file 1A, B.** Intra-operative educational video showing a right spVATS middle lobectomy and mediastinal lymphadenectomy.

## Data Availability

The datasets used and/or analyzed during the current study are available from the corresponding author on reasonable request.

## Abbreviations

VATS: Video-assisted thoracoscopic surgery
spVATS: Single-port video-assisted thoracoscopic surgery
R0: Negative resection margin
PLC: Primary lung cancer
PM: Pulmonary metastasectomy
KHCC: King Hussein Cancer Center
MOT: Mean operative time
MAGS: Magnetic anchoring and guidance system
